# Incidental Coronary Sinus Stenosis During Coronary Angiography: A Rare Angiographic Finding

**DOI:** 10.1155/cric/9568368

**Published:** 2026-06-19

**Authors:** Nazanin Alaei Faradonbeh, Ehsan Khalilipur, Mobina Yarahmadi, Ali Shamsedini

**Affiliations:** ^1^ Department of Emergency Medicine School of Medicine, Iran University of Medical Sciences, Tehran, Iran, iums.ac.ir; ^2^ Rajaie Cardiovascular Medical and Research Center, Iran University of Medical Sciences, Tehran, Iran, iums.ac.ir; ^3^ Physiology Research Center, Iran University of Medical Sciences, Tehran, Iran, iums.ac.ir

**Keywords:** angiography, coronary sinus, heart failure, left ventricular end-diastolic pressure, obstruction

## Abstract

**Background:**

The coronary sinus (CS) is a major venous drainage pathway of the heart but is rarely the site of clinically significant pathology. When obstruction of the CS occurs, it can lead to serious hemodynamic consequences and a high risk of mortality if not promptly identified.

**Case Presentation:**

We report the case of a 60‐year‐old male with Type 2 diabetes mellitus on chronic hemodialysis, who presented with chest pain and dyspnea. While undergoing diagnostic coronary angiography for suspected ischemia, severe stenosis of the CS was incidentally observed. The patient had elevated left ventricular end‐diastolic pressure (LVEDP) and diffuse coronary artery disease. Management included optimization of volume status through intensified hemodialysis, along with guideline‐directed heart failure therapy. The patient′s symptoms and hemodynamic parameters subsequently improved.

**Conclusion:**

This case highlights the importance of recognizing rare CS abnormalities during coronary angiography. Although the clinical significance of incidental CS stenosis remains uncertain, awareness of such findings may help guide further evaluation in selected patients.

**Learning Objective**

• Define coronary sinus obstruction and describe its clinical presentation.
• Explain the causes and risk factors associated with coronary sinus obstruction.
• Identify the diagnostic criteria and imaging modalities used to diagnose coronary sinus obstruction.
• Discuss the management options and potential complications of coronary sinus obstruction.
• Analyze a rare coronary angiographic picture of coronary sinus obstruction and interpret the findings based on the knowledge gained from this educational material.

## 1. Introduction

The coronary sinus is the major vein of the greater cardiac venous system; it has the function of draining deoxygenated blood from myocardium [1].

Previous studies have shown that significant coronary sinus obstruction has been associated with chest pain, dyspnea, ischemic electrocardiogram (ECG) changes, and, in rare cases, sudden cardiac death. The mortality of such a condition is high [2, 3].

Due to the presence of shunts and the special condition of the coronary sinus, partial obstruction does not cause death acutely [4].

Congenital coronary sinus stenosis is a rare malformation that may be detected by echocardiography, multidetector row computed tomography (CT), and intraoperative evaluation [5].

In this article, we introduce a rare case in which cardiac sinus vein stenosis was accidentally found in his angiography.

## 2. Case Report

**Figure 1:**
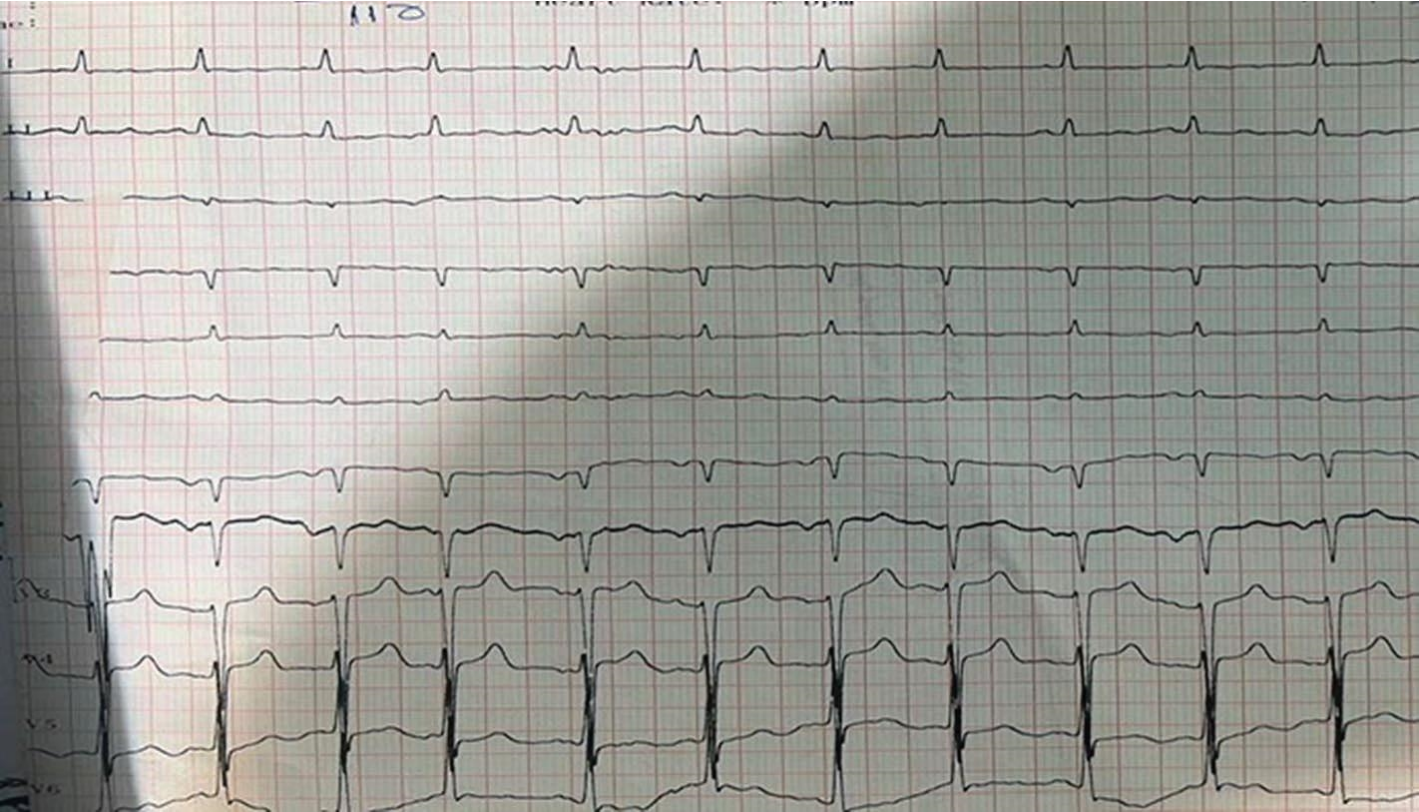
Electrocardiogram.

**Figure 2:**
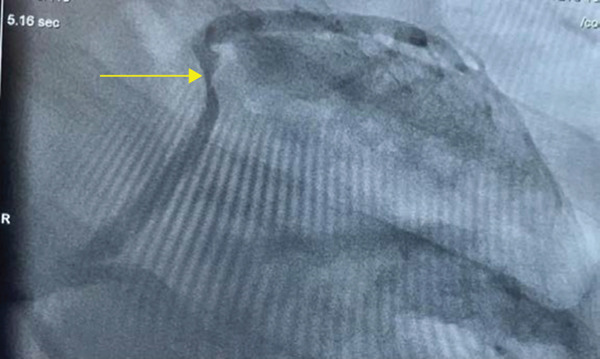
Coronary angiography: severe focal stenosis at the coronary sinus (arrow).

A 60‐year‐old man with a history of Type 2 diabetes mellitus and on hemodialysis presented to Rasoul Akram Hospital, Tehran, Iran, with complaints of chest pain and shortness of breath. His ECG showed signs indicative of cardiac ischemia, including T‐wave inversion in one lead and nonspecific changes in the inferior leads (Figure 1). An echocardiogram revealed a reduced ejection fraction (approximately 35%) and global hypokinesia. Additionally, moderate tricuspid regurgitation (TR) and moderate mitral regurgitation (MR) were observed. Given the reduced ejection fraction and chest pain, coronary angiography was performed. Left ventricular catheterization revealed an elevated left ventricular end‐diastolic pressure (LVEDP), but coronary angiography showed only moderate (60%) diffuse stenosis in the mid‐LAD. However, severe stenosis in the coronary venous system was incidentally noted (Figure 2), which impeded adequate drainage of deoxygenated blood. Severe stenosis of the coronary sinus was incidentally observed during coronary angiography. Elevated LVEDP was also noted; however, the hemodynamic significance of the coronary sinus stenosis could not be definitively established. Importantly, the patient had no history of coronary interventions, prior cardiac surgery, or implantation of a coronary sinus reducer device. In the absence of prior cardiac surgery, coronary sinus instrumentation, or coronary sinus reducer implantation, the stenosis was considered possibly congenital, although an acquired etiology could not be excluded. Considering the congenital nature of this anomaly, the patient was managed conservatively without any interventional procedure, relying instead on adequate hemodialysis and careful fluid removal. As a result, the frequency of hemodialysis was increased to three times per week, and the patient was started on Lasix along with a combination of beta‐blocker and sacubitril/valsartan as per heart failure guidelines. With intensification of dialysis and optimization of fluid balance, the patient′s symptoms progressively improved, confirming that this conservative approach was an adequate and effective treatment strategy.

## 3. Discussion

An increase in the LVEDP is detrimental in two ways including decreased coronary blood flow and increased myocardial oxygen demand [6].

In the presented patient, severe coronary sinus stenosis was incidentally identified during coronary angiography. However, due to the lack of specialized tools for coronary venous flow assessment, the hemodynamic significance of this finding could not be directly evaluated. Elevated LVEDP in this patient may have been multifactorial, potentially related to volume overload, uremic state, reduced myocardial contractility, and underlying cardiac dysfunction. The patient′s clinical improvement following intensified hemodialysis and medical therapy suggests that inadequate volume control likely played a major role in symptom development.

The coronary sinus is a connection of veins that drain the deoxygenated blood carried by the small, middle, great, posterior, and oblique cardiac veins from the myocardium into the right atrium [7].

The coronary sinus receives little attention in literature, because of the few pathological states. However, when the coronary sinus has obstruction, the disease course can be rapidly progressive with a very high rate of mortality [8]. But in this case, the patient survived by careful examination and correct diagnosis. Sometimes obstruction in the coronary vein due to thrombosis or along with other cardiac anomalies may be seen, but isolated severe coronary sinus stenosis without prior coronary manipulation is extremely rare [5].

In such a situation, heart failure and blood pressure lowering drugs are first used, and if the symptoms do not improve, progressive balloon dilatation with a percutaneous coronary intervention (PCI) technique or surgical treatments are used [9]. The mentioned patient has responded to drug treatments.

This case report has several limitations that should be acknowledged. First, the lack of specialized tools to measure coronary sinus flow, such as Doppler ultrasound or intravascular imaging techniques, limited the ability to directly assess the hemodynamic significance of the observed stenosis. Although elevated LVEDP and clinical symptoms suggested impaired venous return, objective measurements would have provided stronger evidence to support this conclusion. Therefore, a causal relationship between coronary sinus stenosis and elevated LVEDP could not be definitively established in this case.

Second, advanced imaging modalities such as cardiac magnetic resonance imaging (MRI), CT angiography, or dedicated venography were not utilized. These techniques could have offered a more detailed anatomical evaluation of the coronary venous system, ruled out other causes of obstruction (such as external compression or thrombosis), and confirmed the congenital nature of the stenosis.

Additionally, as this is a single case report, the findings cannot be generalized to all patients. However, it does highlight an important diagnostic consideration that may be overlooked in standard practice. Clinicians should remain vigilant for anomalies in the coronary venous system during angiographic evaluation, particularly in patients with unexplained elevations in LVEDP, persistent symptoms, or discordance between arterial findings and clinical presentation.

This case reinforces the importance of a comprehensive evaluation of both coronary arterial and venous systems, and it emphasizes the potential impact of rare anatomical variants on cardiac function and clinical outcomes.

Overall, this case highlights an incidental finding of severe coronary sinus stenosis during coronary angiography in a patient with heart failure symptoms and elevated LVEDP. Although the precise hemodynamic significance of the stenosis remains uncertain, recognition of such rare coronary venous abnormalities may be important during routine angiographic evaluation.

## Funding

No funding was received for this manuscript.

## Consent

Before publishing the article, a written informed consent was obtained from the patient in Farsi (the language used by the patient), and the patient gave permission to publish his disease information without publishing his name and personal information.

## Conflicts of Interest

The authors declare no conflicts of interest.

## Data Availability

The data that support the findings of this study are available on request from the corresponding author. The data are not publicly available due to privacy or ethical restrictions.
